# Efficacy of three methods for inserting calcium hydroxide-based paste in root canals

**DOI:** 10.4317/jced.53818

**Published:** 2017-06-01

**Authors:** Thales Galvão, Bernardo Camargo, Luciana Armada, Flávio Alves

**Affiliations:** 1 MSc, Department of Endodontics, Faculty of Dentistry, Estácio de Sá University, Rio de Janeiro, RJ, Brazil; 2 PhD, Department of Endodontics, Faculty of Dentistry, Estácio de Sá University, Rio de Janeiro, RJ, Brazil

## Abstract

**Background:**

To compare the quality of calcium hydroxide paste fillings performed by three different techniques.

**Material and Methods:**

Sixty extracted maxillary central incisors, with previous root canal treatment, were decoronated and the gutta-percha was completely removed from the root canals. Subsequently, the canals were filled with a calcium hydroxide-based paste composed of calcium hydroxide, bismuth carbonate, and glycerin. The study samples were divided into the following three groups on the basis of three insertion techniques (n = 20, each): conventional technique using a hand instrument (MAN), rotary Lentulo spiral (LEN) technique, and a combined technique combining conventional hand files with sonic activation through the EndoActivator device (EA). The quality of fillings was evaluated radiographically by two examiners on the basis of the amount of voids and the apical limit.

**Results:**

The canals filled with LEN or MAN had less void volume compared to the EA technique (*P* >0.01), with no significant differences between them. Considering the apical limits, the three tested techniques showed comparable results (*P* >0.05).

**Conclusions:**

A combined approach utilizing hand files with sonic activation showed no enhancements over the LEN or MAN techniques on the quality of intracanal placement of calcium hydroxide paste.

** Key words:**Calcium hydroxide placement, EndoActivator, Lentulo spirals, intracanal medication, root canal treatment.

## Introduction

Intracanal medication with calcium hydroxide (Ca(OH)2) pastes is commonly used to complement the root canal disinfection after chemo mechanical preparation (1). Studies have shown that this procedural step positively influences the outcomes of endodontic treatment in infected cases (1-3). However, a homogeneous filling up to working length (WL) is essential to ensure adequate effectiveness of Ca(OH)2 pastes (4). Several methods are used to deliver Ca(OH)2 pastes into the root canal, including a hand file, Lentulo spiral, and special devices such as syringes and compactors (5-7).

Studies comparing different techniques to fill root canals with Ca(OH)2 show controversial results. One study found that the endodontic hand file is superior to McSpadden compactor and Lentulo spiral in root canals of dogs premolars, and the endodontic hand file produced the lowest number of empty spaces in the three thirds evaluated (5). However, another study reported that there were no differences in outcomes between using a Lentulo spiral or a hand file in human mandibular premolars (8).

Alternative devices/techniques have been examined to improve the quality of Ca(OH)2 filling. For instance, one study compared different devices/instruments including K-file, ultrasonic file, and Lentulo spiral in single-rooted premolars, and showed comparable quality of fillings (6). Currently, ultrasound as well as sonic devices are very popular among endodontists, and their applications include activation of irrigation solutions (9) and sealer placement (10). Sonic systems are suggested to improve the quality of Ca(OH)2 root canal filling; however, there is no scientific evidence that supports this statement, to date (11).

The aim of this study was to compare the quality of Ca(OH)2 paste filling performed by the following three different methods of application: conventional filling using a hand instrument, filling using a rotary Lentulo spiral, and conventional hand filling complemented by sonic activation. The research hypotheses were: (1) EndoActivator significantly improves the quality of paste filling; and (2) There is no significant difference between the tested techniques.

## Material and Methods

-Teeth selection and initial preparation

Sixty human maxillary central incisors were obtained from a collection of the tooth bank of the University. Reasons for extraction were not related to this study and the research protocol was approved by the institutional ethics committee (CAAE: 0133.0.308.000.10). Teeth were canal-treated by undergraduate students, as part of their pre-clinical training in endodontics.

In all teeth, the filling material was mechanically removed by an LA Axxess #45 (Sybron Dental Specialties Inc., Orange, CA, USA) in the coronal two-thirds and by hand files until a 50 K-file (Maillefer Instruments SA, Ballaigues, Switzerland) 1 mm beyond of the apical foramen. The removal of filling was completed using Hedström files (Maillefer Instruments SA, Ballaigues, Switzerland), in circumferential filing motion, without chemical solvents. The root canals were irrigated with distilled water after each instrument change. At the end of canal preparation, the root canals were dried using paper points. The crowns were then removed using a diamond disc and bucco-lingual and mesio-distal radiographs were obtained to confirm the complete gutta-percha removal.

The apical foramen of each tooth was sealed with a fast setting epoxy resin to create a closed-end system. The study samples were divided into three groups of 20 each. There was no significant difference between groups with regards to the root length (*p* > 0.05). To facilitate the manipulation of samples, the roots were mounted vertically in blocks made of a silicone impression material (President Jet; Coltène AG, Cuyahoga Falls, OH, USA) up to the cervical margin.

A pilot experiment was performed to determine the optimum consistency of the Ca(OH)2 paste for the study. Powder of L & C paste (Dentisply, Petrópolis, RJ, Brazil) (2 g) mixed with glycerin (Hemafarma Comercio e Indústria Farmacêutica, São Gonçalo, RJ, Brazil) (2 ml) was found to have the best consistency. The time of manipulation was 60 seconds. The same mixture was used for all groups and the same operator performed all techniques.

•Manual group (MAN)

A size 50 K-file carrying Ca(OH)2 paste was inserted into the canals up to the apex and removed with counterclockwise movements. This procedure was repeated thrice for each tooth. Thereafter, a cotton pellet was applied gently onto the canal orifice.

•Lentulo group (LEN)

A Lentulo spiral (No. 4, Maillefer Instruments SA, Ballaigues, Switzerland) carrying Ca(OH)2 paste was inserted into the canals up to 2 mm from the root apex. The LEN was powered by a motor (VDW, Munich, Germany), in a clockwise movement at 1000 rpm. Slight pumping motion was applied. This procedure was repeated thrice for each tooth. Thereafter, a cotton pellet was ap-plied gently onto the canal orifice.

•EndoActivator group (EA)

A size 50 K-file carrying Ca(OH)2 paste was inserted into the canals up to the apex and removed with counterclockwise movements followed by application of the EndoActivator (Dentsply Tulsa Dental Specialties, Johnson City, TN, USA) (6) with a blue tip size #35/0.04 positioned at 2 mm from the WL. The EndoActivator was activated at 6.000 cpm for 20 seconds. This procedure was repeated thrice for each tooth. Thereafter, a cotton pellet was applied gently onto the canal orifice.

•Radiographic filling analysis

The roots were radiographed at buccolingual and mesiodistal directions, using occlusal radiographic films (Insight - Kodak Comp, Rochester, NY, USA) with an X-ray unit (Raios-X Timex 70 E, GNATUS, 70 KVp, 7.0 mA, Ribeirão Preto, SP, Brazil). The film-focus distance was set at 15 cm, and the time of exposure was adjusted at 0.4 s. Radiographs were manually processed using the same conditions of time and temperature for the three groups. The radiographs were then digitized using a digital camera (Canon G 9, Canon Inc., Lake Success, NY, USA). The quality of the fillings was evaluated, independently, by two qualified examiners (experienced endodontists), blinded to the techniques used in the experiment. The radiographic images were analysed on the computer screen in a darkened room. Both directions were evaluated for two parameters including the amount of voids and the apical limit of the root canal filling.

For the first analysis, the following scores were used: 1, no voids; 2, presence of voids in less than 25% (< 1/4th) of the root canal area; 3, presence of voids in 25% to 50% (1/4th to 1/2) of the area; 4, voids in more than 50% (>1/2) of area.

The second analysis was based on the apical limit of filling on the two radiographic views. The following scores were used: 1, filling at the apex; 2, filling short 1 to 2 mm from the apex; 3, filling short more than 2 mm from the apex.

The concordance among the examiners was confirmed (kappa values = 0.55 and 0.75, respectively, for detectable voids and apical limit analyses). The worst score was used in cases of disagreement.

•Statistical analysis

Statistical analysis was performed to compare the means obtained by the two examiners. The scores for area of void based on the three filling techniques as well as the teeth length were compared between groups using the Kruskal-Wallis test. The Mann-Whitney test with the Bonferroni’s correction was performed. All statistical tests were performed using the Statistical Package for the Social Sciences (SPSS) software, version 19.0 (IBM, São Paulo, SP, Brazil). A probability level of .05 was used as criterion for statistical significance.

## Results

-Detectable voids

The amount of voids between the tested techniques showed statistical significant difference (*p* < 0.01). In general, EA group showed higher amount of voids, followed by MAN and LEN groups, in order ( Table 1). Score (#1) was not observed in any root. The occurrence of voids was evenly distributed along the canals.

**Table 1:**
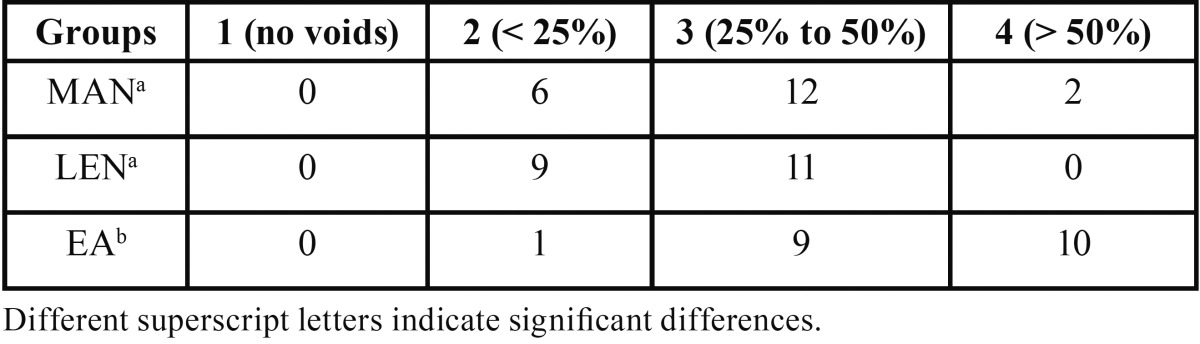
Number of canals per group and the quality of CaOH2 filling regarding the amount of voids (score).

-Apical limit

**Figure 1:**
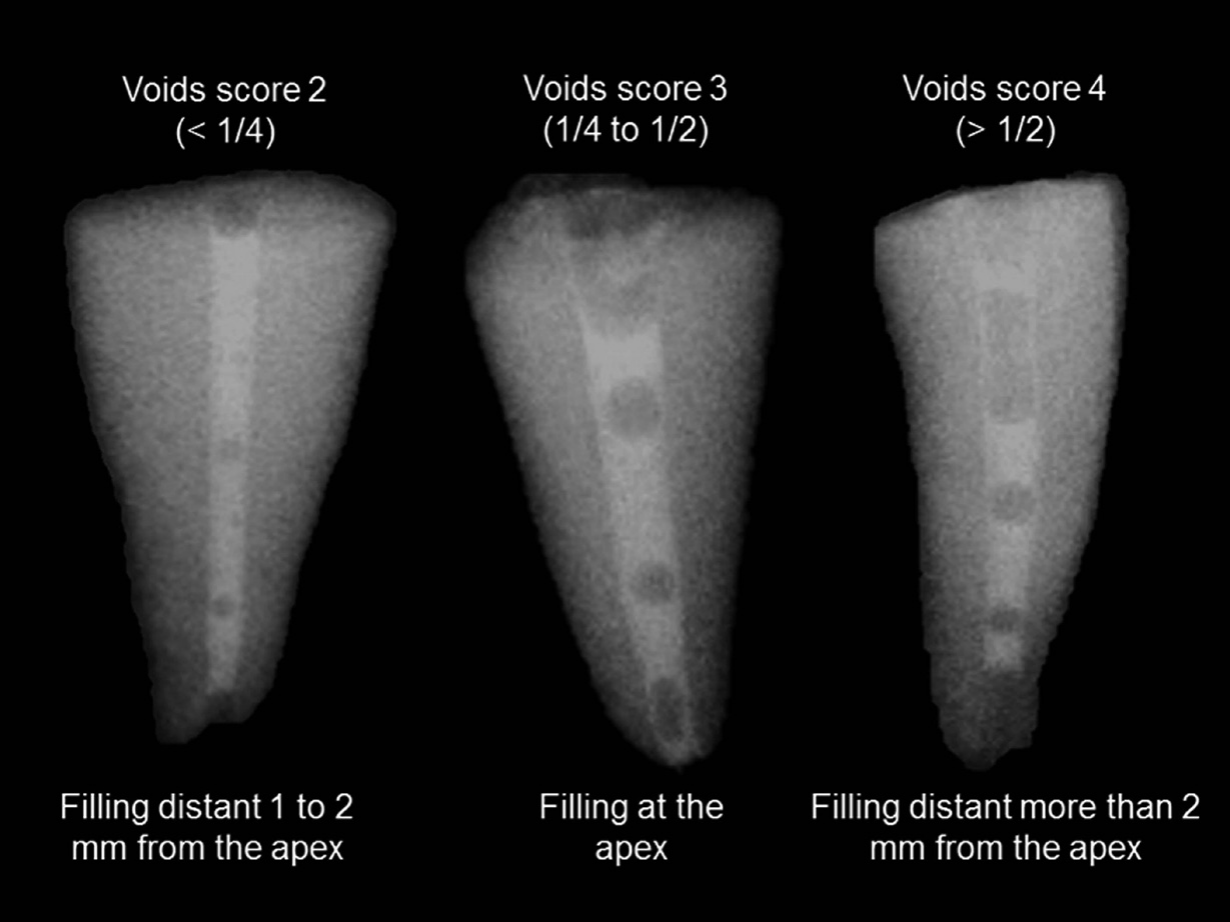
Radiographic filling analysis considering detectable voids and apical limit following the adopted criteria. The better score for detectable voids (#1) was not observed in any root. From the left to the right are specimens from groups: LEN, the technique using rotary Lentulo spiral; MAN, conventional technique using a hand instrument; and EA, a mixed technique combining conventional hand file with sonic activation with the EndoActivator device.

There was no statistical difference between the tested techniques with respect to the apical limit (*p* > 0.05). In general, the root canal filling in LEN and MAN groups was nearest to the apex, as compared to EA group ( Table 2, Fig. 1)

**Table 2:**
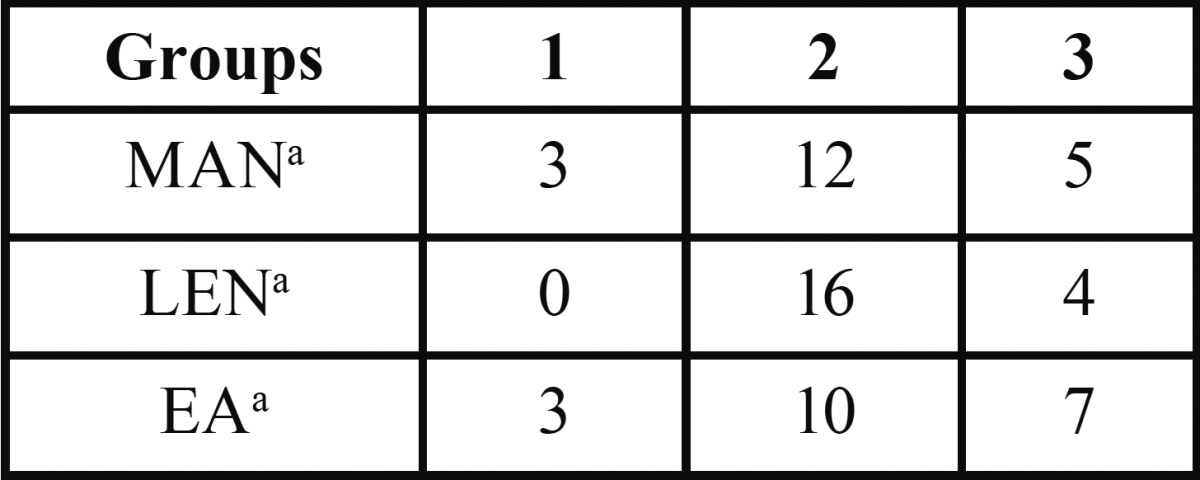
Number of canals per group and the quality of CaOH2 fill regarding the apical limit (score).

## Discussion

Calcium hydroxide paste is the most common intracanal medicament used in endodontics. The biological properties of Ca(OH)2, in particular the antimicrobial action and the repair stimulation by deposition of mineralized tissue are suitable for this application (12,13). To maximize the benefit of their properties, Ca(OH)2 pastes must be in direct contact with the root canal walls and fill the total extension of the main canal space (4).

If the root canal is not well instrumented and irrigated, the medication will not be effective. Therefore, the root canal must be enlarged up to a diameter compatible with its anatomical condition (4,14,15). Regardless of the application mode, filling with medication is not effective in slightly enlarged canals (8). In the present study, the apical preparation was standardized at 0.50 mm for all root canals to guarantee sufficient enlargement for the filling with medication. Additionally, dentin root canals were used in this study only as a tube for medication filling.

In the present study, lentulo spiral was activated at 1000 rpm with an electric motor, as previously reported (16), to avoid any bias related to the speed, which is variable when using compressed air motors. The application of Ca(OH)2 paste with lentulo produced a more homogenous filling followed by manual technique, without statistical difference between them. Moreover, a previous study (17) reported that counterclockwise rotation of K-files did not result in acceptable fillings in root canals enlarged with a #25 file. In the current study, regardless of the application technique, none of the roots showed a better score. Inadequate quality of filling is a special clinical concern in most cases, independent of the insertion technique (score 3 in 53% of cases).

Many studies showed that lentulo spiral was effective in producing homogenous fillings with Ca(OH)2 (16-20), but the effectiveness of EndoActivator for this purpose has not been addressed in previous study. Ca(OH)2 pastes are also available in injectable dispensing forms using special syringes and needles, but the hydraulic pressure required to fill the canals increases the possibility of paste extrusion to the periradicular tissues (21).

The tested techniques were similar in terms of the apical limit, without statistical difference between them. In most cases, the apical limit ranged from 1 and 2 mm from the apex (score 2 in 63% of cases), indicating that none of the tested techniques fully satisfied this requirement. These results corroborate those from a previous study that compared four techniques using Lentulo or Navitip with and without a Ca(OH)2 point inserted into the root canal. The apical portion of root canals was unfilled in all techniques (22). In contrast, an earlier study (17) found that LentuIo spiral was significantly more effective in carrying the paste to WL than counterclockwise rotation of K-files. However, the results of the previous study require careful interpretation, considering the limited number of tested samples.

The technique with EndoActivator was less effective than Lentulo and manual techniques, contradicting the first hypothesis. This application mode was suggested by Ruddle (11), but had not been previously tested. Based on the results of the present study, the use of EndoActivator is contraindicated for this purpose. Sonic agitation seems to dislodge the medication and does not favour the filling.

The present study has some limitations including the use of teeth with non-complicated root canal anatomy, the two-dimensional analysis, and the in vitro experimental design. For these reasons, the results are not directly applicable to the clinical field.

## Conclusions

Considering the limits of this in-vitro study and the fact that the root canals employed presented non-complicated anatomy, is concluded that well performed manual insertion of Ca(OH)2 paste produced similar quality of filling in comparison with Lentulo technique, and both were better than the technique with EndoActivator. However, none root canal was completely filled with the paste and the apical limit was achieved in a few cases, independently of the technique. These statements encouraging the development of more effective Ca(OH)2 filling techniques.
